# Perceived Preparedness of Internal Medicine Interns for Residency and the Value of Transition to Residency Courses

**DOI:** 10.7759/cureus.50868

**Published:** 2023-12-20

**Authors:** Anthony Gaynier

**Affiliations:** 1 Medical Education, Wayne State University School of Medicine, Detroit, USA

**Keywords:** internal medicine residency, perceived quality, residency and internship, transition to internship, medical school education, general internal medicine, transition to residency

## Abstract

The popularity of Transition to Residency (TTR) courses has been increasing in U.S. medical schools. Yet there is limited data on interns’ perceived preparation for residency and the value of nonsurgical TTR courses and their common components. Research has shown that TTR courses increase medical knowledge, clinical skills, and an increase in confidence in participants, but still, incoming residents do not feel prepared for the start of residency. Currently, there are only a few single institution studies researching interns’ perceived value of TTR courses.

This quantitative study surveyed internal medicine interns at five residency programs to find their preparation for residency, their preparedness in TTR competencies, and the perceived value of common TTR components. Data show that IM interns still feel unprepared for residency. The data also indicate that individuals who engage in TTR courses feel more prepared in TTR competencies compared to those who do not participate in TTR courses. Finally, internal medicine interns found the common TTR components valuable.

## Introduction

The goal of medical education is to prepare students for graduation and for the subsequent step, which is residency. Many medical schools utilize a common educational model containing two years of preclinical training in basic sciences, followed by two years of clinical work focusing on putting this knowledge into action while taking care of patients.

During medical school, students must retain large amounts of knowledge and skills to succeed in their clinical rotations, pass standardized exams, meet milestones, and complete Entrustable Professional Activities (EPAs). Over the years, there has been a creation of Transition to Residency (TTR) courses to facilitate the TTR and prepare students for internships. These courses, or *boot camps*, were designed to help bridge the gap between knowledge and skills attained in required clerkship and residency expectations. Courses started in the surgical specialties, teaching hands-on surgical skills, have now been spread across all medical specialties [1-3].

The structure of medical education incorporates several transition phases. Initially, there is the transition from basic science education to clinical education, followed by the shift from the intensive clinical experience in the third year to the more relaxed fourth year. The final transition occurs from graduation to the commencement of residency. Each of these transitions is carefully managed to prepare students for the subsequent phase of the curriculum. With the transitions, as students reach the next stage of their education there is a time where the student feels unprepared for their new level of responsibility [4,5]. The idea of medical education as a continuum instead of rigid building blocks is an idea that would help to ease these transitions and sudden increases in expectations and responsibility. Currently, we are not near this goal of a continuum, but some help with transition has occurred in medical education.

TTR courses were first created by medical schools as elective courses for students pursuing a surgical residency [6]. The course was made to assist in basic surgical skills before students enter the surgical residency. Similar courses have spread across medical schools and studies were done to assess the value of TTR courses in surgical rotations. The literature stated these courses increased knowledge [7], clinical skills [8], and most importantly confidence in medical students [9].

TTR courses have spread across the country and into non-surgical specialties, with over 100 institutions in the American Association of Medical Colleges (AAMC) included in their curriculum [10]. The AAMC does not currently have a standard for these courses, thus the curriculum varies between schools and specialties. Each medical school creates its course, course name, curriculum, goals, objectives, length of course, etc. The literature states that despite the variance in these courses, they do on average increase confidence and clinical skills in medical students entering this time of transition [9].

Sachdeva et al. [11] hypothesized that students entering residency would have different abilities because of differing medical school curriculum and their skills and talents. Residents have long hours, high levels of responsibility, debt, career planning, and low self-confidence are just some of the factors that cause this stressful transition [4]. For the first time, first-year residents or interns are responsible for patients. The combination of stress and responsibilities caused graduating medical students to feel unprepared for residency. TTR courses may help to mitigate this stress and further refresh the skills and knowledge needed to succeed in residency.

Peyre et al. [9] found that surgical interns felt unprepared for residency. This study built on the findings of Sachdeva et al. [11] but went a step further by trying to improve the confidence of interns. Peyre et al. instituted a TTR course for surgical interns, containing specific surgical training for participants. After completion of the course, those who participated were able to improve their confidence in preparedness for residency. Esterl et al. [12] also found a similar improvement in confidence in subjects who completed a TTR course at another institution. Improvement in confidence following a TTR course became a common theme in the literature [12].

Another common theme in TTR courses was the gain of medical knowledge. Boehler et al. [7] studied both surgical interns and students who were interested in pursuing a surgical residency. Both groups underwent a pre- and post-knowledge exam. In the pretest, both medical students and surgical interns had similar scores. The medical students then participated in a TTR course combining both didactic and simulation activities. After this course, the students and interns took a posttest. In the posttest, the medical students who participated in the TTR course scored significantly higher than the surgical interns. Boehler et al. [7] was able to show the importance of TTR courses on medical knowledge. Antonoff et al. [13] studied medical students who did and did not participate in TTR courses. Students were evaluated at the end of July (their first month of internship). Students who participated in TTR courses performed better in July than those who did not participate. Antonoff et al.’s findings showed the value of both medical knowledge and clinical skills after a TTR course [13]. An increase in clinical skills is another theme found after the completion of TTR courses. Scicluna et al. [14] found that after a TTR course, students’ confidence and perceived clinical skills increased. Santen et al. [8] were also able to use a nonsurgical TTR course to increase the clinical skills of students.

TTR courses show an improvement in clinical skills, medical knowledge, and confidence for those who participate. Angus [15] was able to survey residency program directors and identify areas that were important for interns to succeed in residency. These skills included medical knowledge, clinical skills, communication, professionalism, time management skills, and the ability to prioritize. Elnicki et al. [1] studied a common curriculum in the fourth year of medical school. In the analysis, Elnicki et al. identified that medical knowledge, clinical skills, communication, professionalism, time management skills, and the ability to prioritize are common sections of surgical and nonsurgical TTR courses.

TTR courses deliver not only specific medical knowledge and clinical skills but also common sections that can be mapped to deeper competencies. Elnicki et al. [1] studied to find what TTR courses across the AAMC offer their students. In the creation of the TTR course here at Wayne State School of Medicine (WSUSOM), Elnicki et al.’s findings were imperative to the designing of the curriculum. After mapping WSUSOM’s curriculum, it is evident that the main focus of the TTR course is the eight competencies: communication, professionalism, teamwork, decision-making, navigating difficult situations, specific knowledge/skills, efficiency, and consultation. This is similar to the findings of Angus et al. [15], students need these skills to succeed in residency. These, in addition to the specific skills and medical knowledge, allow for the formation of successful interns.

TTR courses show an increase in clinical skills, medical knowledge, confidence, and training in communication, professionalism, teamwork, decision-making, and navigating difficult situations. However, what has not been shown in the literature is what specifically should be included in the TTR curriculum. Varma et al. [16] and Chang et al. [17] were able to study interns to understand what sessions were valuable in TTR courses and what sessions and objectives they wished were included in these courses. Both studies were done at single sites with limited numbers of residents. Varma et al. [16] found that interns would have liked the addition of antibiotics, electrolyte management, and crossover topics to the curriculum delivered at the University of Pittsburgh. Chang et al. [17] used a qualitative study of interns to identify themes and categories relating to the challenges of TTR, identifying possible areas of improvement in the medical school curriculum that could be addressed by TTR courses. This study will integrate findings from the review conducted by Elnicki et al. [1], qualitative themes identified by Chang et al. [17], suggested curriculum additions by Varma et al. [16], and, finally, insights from the existing WSUSOM curriculum. The aim is to achieve a comprehensive understanding of Internal Medicine (IM) interns across various institutions, specifically focusing on what they found valuable in their TTR courses and areas where they express a desire for more training before entering residency. 

In 2020, 40,084 MD and DO graduates applied for 37,256 residency positions in the Main Residency Match®, according to the National Resident Matching Program [10]. Many of these graduates feel unprepared for residency [9]. As many residency programs struggle with unprepared residents, it is critical medical schools increase their ability to train competent and confident graduating medical students [9]. TTR courses have shown the ability to improve confidence in clinical skills, overall preparedness for residency, and medical knowledge and decrease anxiety in graduating medical students. By better identifying interns’ needs, medical schools can improve training and preparation for residency. TTR courses and their components should be an area of consideration for residency orientation.

The purpose of this study is to survey IM interns and interpret their perceived value of nonsurgical TTR courses and their common components sessions. In addition, this study gathers and interprets data regarding their preparedness for residency, preparedness in TTR competencies, stress of internship, and level of burnout. This information may be advantageous for the adaptation and creation of more successful TTR courses and a smoother transition to graduate medical education. 

This study examines data collected from IM interns and their opinions on nonsurgical TTR courses. The first research question is the following: How do IM interns report their preparedness in TTR competencies? The second research question is as follows: To what extent do IM interns value nonsurgical TTR courses’ common components? 

The data collection from interns has the potential to enhance the quality of TTR courses in the future and contribute to the advancement of scholarly research that has already been conducted. With this information, institutions may be able to improve current courses, allow easier creation of new courses, and allow for more scholarly research to assist in the possible standardization of TTR courses.

## Materials and methods

Research questions

This study analyzes data collected from IM interns and their perspectives on nonsurgical TTR courses. The first research question explores how IM interns articulate their preparedness in TTR competencies. The second research question investigates the extent to which IM interns appreciate nonsurgical TTR courses and their common components. Table 1 outlines the survey design for this study.

**Table 1:** Survey design. TTR, Transition to Residency; ANOVA, analysis of variance

Section	Questions	Data	Method of analysis
Section 1	1-7	Survey data regarding demographics:	Descriptive statistics (e.g., mean, standard deviation, min, max)
		Residency program	
		Gender	
		Age	
		Ethnicity	
		Previous career	
		Participation in TTR courses	
Section 2	8-10	Survey data regarding stress level and burnout:	Descriptive statistics (e.g., mean, standard deviation, min, max)
		Current stress level	
		Abbreviated Malsach scale [18]	ANOVA to compare stress and burnout levels by demographic groups.
Section 3	9-23	Survey data regarding preparedness for residency and in TTR Competencies:	Descriptive statistics (e.g., mean, standard deviation, min, max)
		Communication	
		Professionalism	
		Navigating difficult situations	ANOVA to compare the perceived value of TTR competencies by demographic groups
		Teamwork	
		Decision-making	
		Efficiency	Regression analysis - competencies
		Consultant services	
		Ability to perform under stress	
Section 4	24-30	Survey data regarding common TTR components in the literature:	Descriptive statistics (e.g., mean, standard deviation, min, max)
		Elinicki et al. [1]	
		Varma et al. [16]	
		Chang et al. [17]	Regression analysis - composite variables from Elnicki et al [1]
		Wayne State University School of Medicine (WSUSOM) TTR curriculum	

Research design and setting

A descriptive survey research design was used in this study. Data were collected by the researcher using an online survey tool (Qualtrics, Provo, UT). This study is quantitative in nature. The original data collection was completed at IM residency programs in five Metro-Detroit area hospitals, namely, Detroit Medical Center (DMC) Detroit Receiving Hospital, Detroit Medical Center Sinai Grace Hospital, Henry Ford Hospital, Beaumont Hospital Dearborn, and Authority Health Internal Medicine Residency Program, which serves multiples hospitals, unlike the other programs. These residency programs have a relationship with WSUSOM and have volunteered to be a part of this descriptive survey.

Participants

A total of 64 interns are part of the IM residency programs at the included institutions as of January 2023. Of these, 64 out of 129, representing a response rate of 50%, completed the survey. The inclusion criteria for this study require participants to be first-year residents (interns) at the following metro-Detroit area hospitals with an affiliation with WSUSOM. This study was voluntary, and any intern from these programs was eligible to participate, regardless of the location of their undergraduate medical education. All data obtained from the survey are without any identifiers on individual participants. The residency programs included in the study varied in size, with the following participant numbers for each program: Detroit Medical Center Detroit Receiving Hospital (*n* = 29, 45.3%), Detroit Medical Center Sinai Grace Hospital (*n* = 15, 23.4%), Henry Ford Hospital (*n* = 14, 21.9%), Beaumont Hospital Dearborn (*n* = 4, 6.3%), and Authority Health Internal Medicine Residency Program (*n* = 2, 3.1%). The residency programs are located in Metro Detroit but serve different hospital systems, hospitals, different patient populations, etc.

Surveying multiple residency programs with varying intern sizes, locations, and patient populations makes the sample more generalizable. Multi-institution is done purposefully to have deliberate heterogenous sampling on presumptuously important dimensions is an important strategy for generalization. Adding to the sample size can enhance generalization, even when samples are not random, the more replicates there are, the greater the likelihood that the unusual cases will cancel each other out [19].

Instrument

This study employs a quantitative convenience sample survey design to assess overall preparedness for residency, specifically focusing on preparedness in the TTR competencies, skills, and knowledge deemed crucial for success in residency. Additionally, it explores participants' identified areas where they desire more training, as well as their stress levels, burnout experiences, and demographic information through a series of questions. This study bases its survey design on the works of Elnicki et al. [1], Varma et al. [16], and Chang et al. [17], along with incorporating insights from the current curriculum of WSUSOM TTR

Section 1 addresses student background attributes (residency program, age, gender, race, ethnicity, previous career, and participation in the TTR course) or the independent variables, which are spoken about in more detail later in the paper. Section 2 investigates the stress and burnout of IM interns, using questions from Lim et al. [18]. Section 3 investigates interns’ perceived value of TTR competencies. These competencies are mapped from the WSUSOM in Table 2 but are the main goals for students to learn in TTR courses. The TTR competencies are Communication, Professionalism, Teamwork, Decision Making, Navigating Difficult Situations, Specific Skills/Knowledge, Efficiency, and Consultation. Section 4 addresses the TTR common components from the study done by Elnicki et al. [1]. In addition, it includes the suggestions taken from studies by Chang et al. [17] and Varma et al. [16] that surveyed residents and the value of specific TTR courses at single sites. 

**Table 2:** TTR competencies. TTR, Transition to Residency

Session	Competency 1	Competency 2	Competency 3	Competency 4	Competency 5	Competency 6	Competency 7	Competency 8
Chief Medical Resident Expectations	Communication	Professionalism	-	-	-	Specific Skills/Knowledge	-	-
Documentation	Communication	Professionalism	Teamwork	-	-	Specific Skills/Knowledge	Efficiency	-
Nurse Cases	Communication	Professionalism	Teamwork	Decision-Making	Navigating Difficult Situations	Specific Skills/Knowledge	Efficiency	-
Quality Improvement	Communication	-	-	-	-	Specific Skills/Knowledge	-	-
Social Determinants of Health	Communication	-	Teamwork	Decision-Making	-	Specific Skills/Knowledge	Efficiency	-
Death	Communication	Professionalism	-	Decision-Making	Navigating Difficult Situations	Specific Skills/Knowledge	Efficiency	-
Teaching	Communication	Professionalism	Teamwork	-	-	Specific Skills/Knowledge	-	-
Common Problems	-	-	-	Decision-Making	-	Specific Skills/Knowledge	Efficiency	-
Nurse Cases #2	Communication	Professionalism	Teamwork	Decision-Making	Navigating Difficult Situations	Specific Skills/Knowledge	Efficiency	-
Clinic Tips	Communication	Professionalism	Teamwork	-	-	Specific Skills/Knowledge	Efficiency	-
Sign-Outs	Communication	Professionalism	Teamwork	-	-	Specific Skills/Knowledge	Efficiency	-
Writing Orders	Communication	Professionalism	Teamwork	-	-	Specific Skills/Knowledge	Efficiency	-
Whose Line Is It Anyway	-	-	-	Decision-Making	-	Specific Skills/Knowledge	Efficiency	-
Team Dynamics	Communication	Professionalism	Teamwork	-	-	Specific Skills/Knowledge	-	-
COVID-19 and Common Problems	-	-	-	-	-	Specific Skills/Knowledge	Efficiency	-
Telehealth	Communication	Professionalism	-	-	-	Specific Skills/Knowledge	Efficiency	-
Blood Products	-	-	-	-	-	Specific Skills/Knowledge	Efficiency	-
Code Gray	Communication	Professionalism	Teamwork	Decision-Making	Navigating Difficult Situations	Specific Skills/Knowledge	Efficiency	-
Crucial Conversations	Communication	Professionalism	Teamwork	Decision-Making	Navigating Difficult Situations	Specific Skills/Knowledge	Efficiency	-
Mental Health First Aid	Communication	-	-	Decision-Making	Navigating Difficult Situations	Specific Skills/Knowledge	-	Consultation
When to Consult Psychiatry	Communication	Professionalism	Teamwork	Decision-Making	Navigating Difficult Situations	Specific Skills/Knowledge	Efficiency	Consultation
Communication With the Emergency Department	Communication	Professionalism	Teamwork	Decision-Making	Navigating Difficult Situations	Specific Skills/Knowledge	Efficiency	Consultation
Central Line and Pronouncing Death	Communication	Professionalism	Teamwork	Decision-Making	Navigating Difficult Situations	Specific Skills/Knowledge	Efficiency	-

The instrument is an online quantitative survey developed on Qualtrics and distributed to Program Directors/Coordinators of WSUSOM-affiliated IM Residency Programs. The survey is intended for completion by the programs' interns. This survey methodology is consistent with the studies referenced in this paper. Recently, Varma et al. [16] and Chang et al. [17] used online surveys completed by interns to add to TTR literature.

Scoring

In section 1, demographic data were collected. These represent nominal variables creating comparison groups. In section 2 and section 3, the responses to the items were recorded and a mean was calculated from the responses. In addition, composite variables were created using Cronbach’s alpha to measure the correlation between questions. This was done for Section 4 of the survey (TTR common components), grouping them in the way Elnicki et al. [1] grouped them from the literature search they completed.

Reliability and validity

The majority of this survey was adapted from other surveys, including the modified Maslach’s burnout scale [18], and the questions regarding stress, which both have been extensively tested for reliability and validity using Cronbach’s alpha and factor analysis. In addition, the questions on the survey are measured in a Likert scale using questions from Elnicki et al. [1] and Varma et al. [16], which all had been tested for reliability and validity. A pilot group was created to ensure further reliability and validity. 

Data collection procedures

Data collection was completed using Qualtrics, an online survey system used at WSUSOM. The survey link was disseminated on January 4, 2023, and closed on March 4, 2023. This allowed two months for survey completion. The months of January-March were chosen to give IM interns a time of transition and understanding of residency. Interns commonly commence their duties on July 1. This time gap allows interns to transition into their roles while retaining the recollection of their feelings before entering residency. Emails were sent out by Program Directors and Coordinators to assist in the completion of the survey, following the Institutional Review Board (IRB) guidelines. On January 10 and February 20, Program Directors and Coordinators sent reminder emails to interns for completion. 

Data analysis

The data obtained from the surveys were analyzed using IBM SPSS Statistics for Windows, Version 28.0 (IBM Corp., Armonk, NY). The data analysis was divided into four sections. Section 1 included demographic questions (independent variables). The section compared differences between groups and relationships with the TTR competencies and common curriculum.

In section 2, data were collected on the stress and burnout levels of interns using data from a study by Lim et al. [18]. Section 3 of the analysis used descriptive statistics to provide baseline information on the eight TTR competencies referenced in Table 2 and skills taught in TTR courses. Section 4 of the survey gathers data on previous research done by Elnicki et al. [1], Varma et al. [16], Chang et al. [17], and the WSU TTR curriculum.

In section 3, ANOVAs were conducted to compare the residency program, age, gender, race, ethnicity, previous career, and participation in TTR course (independent variables) to IM intern’s responses to stress/burnout, TTR competencies, and common TTR course components found by Elnicki et al. [1] (dependent variables). In addition to conducting ANOVAs, t-tests were used to assess differences in mean Perceptions of Preparedness in Competencies. Similarly, t-tests were conducted on the composite variables of common TTR components to compare individuals who participated in the TTR course with those who did not. Finally, regression analysis was completed to compare demographic groups with composite TTR common components to identify any relationships between variables. The survey and composite variables and how they were created are given in Appendix.

Table 3 shows the descriptive statistics for the research study. 

**Table 3:** Descriptive statistics. *N*, number of participants; Std. dev, standard deviation; Min, minimum; Max, maximum; TTR, Transition to Residency; DMC, Detroit Medical Center; SGH, Sinai Grace Hospital

Variable	N	Mean	Std. dev.	Min	Max
TTR participation	64	0.42	0.50	0	1
DMC	64	0.45	0.50	0	1
SGH	64	0.23	0.43	0	1
HFHS (Henry Ford Health)	64	0.22	0.42	0	1
Beaumont	64	0.06	0.24	0	1
Authority health	64	0.03	0.18	0	1
Female	64	0.44	0.50	0	1
White	64	0.23	0.43	0	1
Black or African American population	64	0.16	0.37	0	1
Asian	64	0.38	0.49	0	1
Other ethnicity	64	0.23	0.43	0	1
Prior career	64	0.19	0.39	0	1
Stress and burnout					
Stress	61	5.21	2.10	0	10
Well and whole	61	3.79	1.13	1	5
Callous	61	1.56	1.51	0	5
Burnout	61	2.38	1.38	0	5
TTR competencies					
Overall	60	3.32	1.14	1	5
Communication	60	4.08	0.93	1	5
Professionalism	60	4.32	0.95	1	5
Teamwork	60	4.35	0.88	2	5
Decision-making	60	3.02	1.11	1	5
Difficult situation	60	3.23	1.17	1	5
Efficiency	60	3.38	1.28	1	5
Consult	60	3.78	0.92	2	5
Responsibility	60	3.40	1.33	1	5
Function under stress	60	3.72	0.98	2	5
TTR common components					
Procedural	56	4.26	0.78	1.00	5
Medical problem	58	3.89	0.81	1.00	5
Basic science	58	3.94	0.78	1.00	5
Hospital	56	4.33	0.66	1.90	5
Communication skills	56	4.36	0.63	2.33	5
Life skills	55	4.22	0.66	2.22	5

Variables

In quantitative research, the characteristics of the individuals being measured are described as the independent variables [20]. The independent variables for this study consist of student background attributes (residency program, age, gender, race, ethnicity, previous career, and participation in TTR courses). Table 4 shows the dependent variables are the outcomes that are influenced by the independent variables [20]. The dependent variables in this study are IM interns’ perceived value of TTR competencies, overall preparedness, and perceived value of common TTR components.

**Table 4:** TTR course participation by residency program/hospital. TTR, Transition to Residency; DMC, Detroit Medical Center

Hospital	Yes	No
DMC Detroit Receiving	13	16
Sinai Grace Hospital	6	9
Henry Ford	7	7
Beaumont Dearborn	1	3
Authority Health	0	2

Ethical statement

Data collected were deidentified, with no way of tracking back to the subject. This study was approved by the Wayne State University IRB (#22-06-4758).

## Results

The purpose of this study is to examine IM interns and interpret their perceived value of nonsurgical TTR courses and their common component sessions. In addition, this study gathers and interprets data regarding their preparedness for residency, preparedness in TTR competencies, the stress of internship, and the level of burnout and examines any relationships within groups of IM interns.

Two research questions were developed for this study. Each can be answered using descriptive statistics but is strengthened by using inferential statistics to compare residency programs, age, ethnicity, previous career, and participation in TTR courses. All decisions on the statistical significance were made using a criterion alpha level of 0.05. Table 4 introduces the TTR course participation by residency program/hospital.

Participants rated their perceived preparedness overall and in TTR competencies on a five-point Likert scale. Overall preparedness for the residency by all participants was µ = 3.32. ANOVAs and t-tests were conducted to identify significant differences between demographic groups regarding their overall preparedness and preparedness in TTR competencies. However, no significant differences were found between the groups. There was no significant difference (*P* < 0.05) observed between those who participated in a TTR course and non-TTR course participants, despite the presence of some interesting findings.

As indicated in Table 5, individuals who took part in TTR courses express a slightly lower sense of overall preparedness compared to those who did not participate, although the difference is not statistically significant (*P* < 0.05). TTR course participants felt more prepared for 7/9 TTR competencies than nonparticipants. In the specific TTR competencies of Communication, Professionalism, and Teamwork, both TTR participants and nonparticipants reported feeling adequately prepared for residency. As for the remaining TTR competencies, there is still a need for improvement in perceived preparedness.

**Table 5:** t-Tests for differences in mean perceptions of preparedness. *P* < 0.05.

Preparedness area	TTR course participant mean	Non-TTR course participant mean	*P*-value for t-stat
Overall Preparedness	3.25	3.42	0.5842
Communication	4.14	4.00	0.5736
Professionalism	4.47	4.08	0.1203
Teamwork	4.42	4.25	0.4768
Decision-Making	3.19	2.75	0.1305
Difficult Situation	3.22	3.25	0.9291
Efficiency	3.53	3.17	0.2870
Consult	3.78	3.79	0.9550
Responsibility	3.47	3.29	0.6107
Function Stress	3.81	3.58	0.3921

TTR components were measured on a five-point Likert scale. The study participants agreed that the common TTR components should be included within a TTR course (ranging from basic science, µ = 3.94, to communication skills, µ = 4.36). t-Tests and ANOVAs were completed to compare groups. Significant differences (*P *< 0.10) were identified when comparing perceived values of common TTR components when comparing TTR course participants and non-TTR course participants. This information can be found in Table 6. Those who did not participate in the TTR course rated the hospital and Communication common components as significantly more valuable than those who participated in a TTR course. 

**Table 6:** t-Tests for differences in mean perceptions of the value of TTR common components. ^*^P < 0.10. ^**^P < 0.05. ^***^P < 0.01. TTR, Transition to Residency

Preparedness area	TTR course participant mean	Non-TTR course participant mean	*P*-value for t-stat
Procedural	3.98	4.45	0.0501
Medical Problems	3.59	4.09	0.0360
Basic Science	3.63	4.13	0.0352
Hospital	3.99	4.55	0.0066*
Communication	4.00	4.59	0.0021**
Life Skills	3.91	4.42	0.0098*

Summary

The results of the quantitative data analyses were used to describe the sample and address the research questions posed for the study. Descriptive statistics show that there is room for improvement in overall preparedness for residency and preparedness in TTR competencies. Data analysis shows that those who did not participate in a TTR course feel more prepared overall for residency than those who did participate, though not significantly. TTR participants felt more prepared in TTR competencies than non-TTR participants. Descriptive statistics also show that common TTR components are valued by IM interns. Non-TTR participants valued common TTR components higher, some significantly more, compared to those who participated in TTR courses.

## Discussion

Transitions are a part of life and in medical education. The main focus of undergraduate medical education is to prepare students to graduate and become successful residents. A strategy to assist in doing this goal is the inclusion of TTR courses into the medical education curriculum. TTR courses began in surgical specialties, preparing students to do more hands-on preparation in clinical skills; currently, there are over 100 TTR courses in several different specialties. The literature states that these courses increase confidence, improve clinical skills, and increase medical knowledge. As mentioned earlier, there is a need to enhance transitions in medical education. When students progress to the next stage of their education, there is a period during which they may feel unprepared for the increased level of responsibility [4]. O’Brien et al. also stated how the creation of a unified medical education continuum would benefit students: “Each learner has a unique constellation of strengths and weaknesses. When framed as a true continuum, each learner’s progression through medical education should map to a developmental trajectory. Efforts to define and align milestones and EPAs across the continuum are under way in some specialties, but much work remains-including the development of robust longitudinal, performance-based processes of assessment” [21].

Obrien et al. believed the inclusion of TTR courses into more medical schools would assist with their vision for medical education. Both the TTR Educator group and DiMarino et al. [22] are working to give recommendations to assist in the continuum. The TTR Educator group is working on a compendium of TTR resources for medical schools looking to create or improve their courses. DiMarino et al. [22] gave limited recommendations for IM TTR courses, another step in helping form a continuum and standardization of TTR courses. These courses provided value to graduating medical students before graduation. Yet, there is limited information on what interns perceive their value to be after spending some time in residency. In addition, each medical school has its curriculum for TTR courses.

In the review of TTR courses, Elnicki et al. [1] listed over 50 common components included in curricula. These components include the following competencies that are critical in a student’s transition to residency: communication, professionalism, teamwork, decision-making, navigating difficult situations, specific knowledge/skills, efficiency, and consultation. Furthermore, there are limited data on what common components interns perceive as valuable and limited data on interns’ perceived preparation in these competencies upon graduation from medical school. Only Varma et al. [16] and Chang et al. [17] offered data on perceived value from interns, yet these were both done from a single residency site.

This study and specifically the research questions were created to begin to address some of these gaps in the literature. This study was conducted as multiple residency programs and aimed to dig deeper into the preparation of the convenience sample of interns’ perceived preparation in TTR competencies and their perceived value of common TTR components.

Medical education and TTR courses use experience-based learning (EBL) and experiential learning theory (ELT) as a basis for their educational model. Using these educational theories is a step in the right direction with medical education. TTR courses strengthen the tools that students need to function in residency, and these tools are the TTR competencies.

Research question 1 focused specifically on the TTR competencies and interns’ perceived preparation in these competencies. The survey studied overall preparation in residency and then overall preparation in each of the TTR competencies. The mean for overall preparedness in residency was 3.32 on a five-point Likert scale, indicating that participants, on average, do not feel fully prepared for residency. This finding aligns with existing literature [4,5,9]. While TTR courses may contribute to confidence-building and a sense of preparedness for residency, there is still no consensus on the perceived level of preparedness. The data analysis showed that those who participated in a TTR course rated their overall preparedness for residency lower than non-TTR course participants. Yet, in terms of TTR competencies, participants rated their preparedness higher than nonparticipants. This finding was interesting, as one would expect those who feel confident in competencies to feel more prepared overall. This may be because covering a large amount of material in a short amount of time may increase the stress for students, causing them to become aware of the areas in which they may not feel confident. The TTR competencies do give hope that TTR courses are indeed preparing our students in the correct skill areas. The comparison between TTR participants and nonparticipants again was not significantly different. It is possible that this sample size was too small and that this interesting finding may not hold with a more generalized sample. This study was the first to identify the TTR competencies.

Research question 2 focused specifically on the common components of TTR courses. Due to Elnicki et al.’s [1] literature review, along with contributions from Chang et al. [17], Varma et al. [16], and WSU’s TTR curriculum, this section of the study incorporated 57 questions assessing the perceived value of each component of a TTR course. All common components received a score on a five-point Likert scale, none being rated below (µ = 3.09). This shows that despite the different curricula across medical schools and the lack of standardization in the TTR curriculum, no common component scored below 3 on a five-point Likert scale. From Elnicki et al.’s [1] study, composite variables were created and used to identify further relationships between groups, rather than comparing each of the individual TTR components for relationships. The composite variables were created after using Cronbach alpha analysis. Non-TTR participants found these components more valuable than those who did participate in TTR courses, significantly in Hospital and Communication. TTR participants rated the value of common TTR components significantly lower than nonparticipants. The finding is that the common components of TTR courses seem to be valuable. It makes sense that TTR participants who recently were subject to the components of TTR courses would find these less valuable than nonparticipants. TTR participants also stated they were more prepared in TTR competencies, which came from mapping out the TTR common components. With increased preparation, it is possible that individuals no longer perceive the same level of value in these sessions, having already acquired the knowledge and experience from these components. Overall, the absence of any TTR component rated below 3 on a five-point Likert scale is an important finding, indicating that TTR courses are offering a relevant curriculum for participants in the TTR.

TTR courses have meaningful and helpful curricula being delivered to help students in their transition to residency. This part of the study was unique. This is one of the first studies to look at the specific components of TTR courses and the interns’ perceived value at multiple residency programs. Looking at intern perceptions focuses on an important stakeholder - the intern. This can further strengthen the body of knowledge on TTR courses and may help in pushing to further implement and develop TTR courses.

It is obvious in the data that the TTR competencies that interns felt prepared for aligned with the TTR common components that they found valuable. This finding further strengthens the value of TTR courses in their present state. This also shows that the components of this course are valuable to the curriculum of TTR courses, the fourth year of medical school, and as a part of undergraduate medical education. The competencies for which interns did not feel prepared were making decisions with limited knowledge (µ = 3.02), navigating difficult situations (µ = 3.23), efficiency (µ = 3.38), and taking full responsibility for patients (µ = 3.4).

When mapping the WSU TTR curriculum, every session had a goal of teaching students specific skills and specific medical knowledge; 18 sessions were mapped to being efficient, 12 for decision-making, and 9 for navigating difficult situations. Because medical students lack experience in being responsible for their patients, it is understandable that students perceived preparation to be lacking in these TTR competencies. There are only a few common components of TTR courses that coincide with the competency of taking full responsibility for patients. Feeling confident in this area takes time and experience; a few sessions in a TTR course cannot fully prepare them for this but instead can offer insights into what to expect in residency. Focusing sessions on these areas of perceived weaknesses may close the gap between confidence and preparation in these areas.

In section 3 of the survey, interns were asked what TTR competency was most important in residency. The top three skills were efficiency, communication, and teamwork. Teamwork and communication are areas in which interns who participated in this study felt that they were prepared. Efficiency is an area where they stated they lacked preparedness. Workload and responsibility increase in residency, and interns are bombarded with requests as they work to fully take care of patients. This is a stark change from medical school where students are not fully responsible for the well-being of the patients. This is one of the areas mentioned that causes stress within transition [4]. This is also an area where students have limited experience, as discussed before, only the sub-internship allows students an increased workload and responsibility within the medical school curriculum. This lack of experience and increased workload make for the need for an intern to be efficient. As discussed earlier, there is a focus of TTR courses on efficiency, including sessions on time management, documentation, and work-life balance. In addition, all sessions introduce students to specific skills and knowledge of situations they may face in residency. Simulation and introduction to these areas also should assist in efficiency when residents face these situations.

When working on the inferential statistics in this study, namely, comparing groups (program, race, gender, prior career, TTR course participation, and TTR length) to the TTR competencies, it was a relief that there were no significant differences between different groups. This shows that TTR courses are unbiased regarding who they benefit. The goal of the curriculum is to help all students gain the knowledge and skills they need, not just specific groups. This is promising, as there is a push to remove longstanding biases in medical education. Once again, the TTR courses and their common components can be seen as a positive in medical education.

This study did not investigate the comparison of the performance of those who participated versus those who did not participate in TTR courses. Instead, the focus was on the preparedness of interns overall and in TTR competencies and the value of common TTR components. This study has added to the body of knowledge by filling a gap in the literature by surveying interns' perceived value of TTR courses and, more specifically, investigating the value of what is commonly taught in these courses. In completion of this study, TTR courses again have been seen in a positive light. The components included in the courses are perceived as valuable. This is fitting, as components use ELT and EBL to help improve confidence, clinical skills, knowledge, and perceived preparation in an internship. 

Implications for research, policy, and practice

Medical educators could see this study as an addition to a strong body of knowledge in support of the implementation and continuation of TTR courses within medical education. In all curricula, there should be quality improvements made to further strengthen the curriculum and keep it relevant to the ever-changing world of healthcare. As stated earlier, there are already more than 100 AAMC institutions that have TTR courses as part of their curriculum because of the benefits of increased confidence, clinical skills, and medical knowledge stated in the literature. This study adds to the benefits, stating that the common components are valuable to IM interns. In addition, this study shows that there are still gaps in medical education and TTR courses specifically. Medical educators can push learners and improve courses to further prepare graduating medical students for preparedness for residency and in preparation for the TTR competencies.

 As stated earlier, there is a push by the TTR Educator group to share TTR materials across institutions, with the hope of finding best practices and someday a standardization of objectives and goals of TTR courses. The TTR Educator group, through their engagement with the work of DiMarino et al. [22], is advancing national conversations on the development and improvement of TTR courses. In the future, like other areas in medical education, there needs to be more than recommendations. A solution would be the creation of standards for TTR courses; these should include the inclusion of a TTR course in the medical school curriculum, an inclusion of common curricular topics, and more deliberate practice on key TTR competencies. These standardizations would be beneficial in graduating medical students feeling more prepared for residency.

Limitations of the study

The generalizability of this study could be influenced by the following factors: the study employed a convenience sample drawn from residency programs associated with WSUSOM and the researcher. The data from this study were only from 64 interns in the Metro-Detroit area. The study data were limited to only 2023 results. This study was completed six months after the beginning of the internship, allowing for perceived preparedness to change over time since the beginning of the internship. The study obtained perceived information from the interns, resulting in response bias. While the participants were expected to respond honestly, they might have answered in ways that reflected what they thought the researcher wanted.

Recommendations for further research

The following recommendation for further research can be used to broaden the understanding of TTR courses and their benefit to medical students, residents, and the medical education community.

Some recommendations include expanding similar surveys to interns to add to the body of knowledge and increase intern/resident stakeholders in TTR courses, increasing the number of participants in the survey to increase the generalizability of the findings, using a longitudinal research design to examine changes of intern perception of TTR competencies and their common components over residency, provide intern evaluations at the time of the survey to compare residency evaluations and survey results, and provide student medical school evaluation data to compare medical school performance to survey results.

## Conclusions

The literature states that TTR courses increase confidence, clinical skills, and medical knowledge, yet medical students still feel unprepared for residency. The data from this study align with the literature, indicating that students still experience a sense of being unprepared for residency. This study contributes to the existing literature by revealing the mixed perceived value of TTR competencies among IM interns. Participants reported feeling prepared in some areas, particularly those where they have gained further experience. However, they expressed a sense of being unprepared in other areas, such as medical knowledge, navigating difficult situations, efficiency, and assuming full responsibility for patients. This study also demonstrated the strong perceived value of the common components of TTR courses. TTR courses add value to the medical education curriculum and continuum. Further development and implementation of these courses could provide continued value in the preparation of medical students entering residency.

## Appendices

Survey tool

Research Information Sheet Title of Study: Internal Medicine Interns' Perceived Preparation for Residency and Perceived Value of Non-Surgical TTR Courses Principal Investigator (PI):                 Anthony Gaynier, MS Wayne State University Purpose: You are being asked to participate in a research study focused at helping medical students with the transition to residency because you are an intern. This study is being conducted at Wayne State University, Detroit Receiving Hospital, Sinai Grace Hospital, Henry Ford Hospital, Beaumont Dearborn Hospital, and the Authority Health IM Residency Program. There are minimal foreseeable risks. Study Procedures If you take part in the study, you will be asked to complete the following survey regarding common topics in Transition to Residency courses, their value, and questions regarding demographic information, stress and burnout.  The survey should take less than 10 minutes. Benefits: As a participant in this research study, there will be no direct benefit for you; however, information from this study may benefit other people now or in the future. Risks: There are no known risks at this time to participation in this study. Costs: There will be no costs to you for participation in this research study. Compensation: You will not be paid for taking part in this study. Confidentiality: All information collected about you during the course of this study will be kept without any identifiers. Voluntary Participation /Withdrawal: Taking part in this study is voluntary. You may choose not to take part in this study, or if you decide to take part, you can change your mind later and withdraw from the study. You are free to not answer any questions or withdraw at any time. Your decision will not change any present or future relationships with Wayne State University or its affiliates.

Questions

If you have any questions about this study now or in the future, you may contact Anthony Gaynier one of his research team members at the following phone number 734-755-1893. If you have questions or concerns about your rights as a research participant, the Chair of the Institutional Review Board can be contacted at (313) 577-1628. If you are unable to contact the research staff, or if you want to talk to someone other than the research staff, you may also call the Wayne State Research Subject Advocate at (313) 577-1628 to discuss problems, obtain information, or offer input. Participation By completing the questionnaire, you are agreeing to participate in this study.

Q1 My name is Anthony Gaynier, I am a student in the Wayne State University Educational Leadership and Policy Studies doctoral program.  I am in need of assistance in gathering data on Transition to Residency (TTR) Courses.  As an Internal Medicine Intern, your responses are crucial to the development of these courses as the number of these courses across the nation is growing.  If you are willing, please proceed to the survey following.  The survey should take no longer than 10 minutes.

Thank you for your assistance, Anthony Gaynier

Q2 Residency Program

o DMC Detroit Receiving  (4)

o Sinai Grace Hospital  (5)

o Henry Ford  (6)

o Beaumont Dearborn  (8)

o Authority Health  (9)

Q3 Gender

o Man  (9)

o Non-binary  (10)

o Woman  (11)

o Prefer to self describe  (12) __________________________________________________

Q4 Age

________________________________________________________________

Q5 Ethnicity

▢         White  (9)

▢         Black or African American  (10)

▢         American Indian or Alaska Native  (11)

▢         Asian  (12)

▢         Native Hawaiian or Pacific Islander  (13)

▢         Other  (14)

Q6 Did you have a previous career before going into medicine?

o Yes  (1)

o No  (3)

Q7 I participated in a transition to residency course

o Yes  (1)

o No  (2)

Display This Question:

If I participated in a transition to residency course = Yes

Q136 Was this Transition to Residency course required?

o Yes  (1)

o No  (2)

Display This Question:

If I participated in a transition to residency course = Yes

Q137 What was the length of the Transition to Residency Course?

o Less than 1 week  (1)

o 1-4 weeks  (2)

o 4+ weeks  (3)

End of Block: Demographics

Start of Block: Stress/Burnout

Q8 How stressed do you feel on a daily basis?

0

1

2

3

4

5

6

7

8

9

10

stress level ()

Q9 Overall, I feel well and whole (emotionally, physically, etc.)

o Strongly agree  (4)

o Somewhat agree  (5)

o Neither agree nor disagree  (6)

o Somewhat disagree  (7)

o Strongly disagree  (8)

Q10 I have become more callous toward people since I started residency

o Never  (1)

o A few times a year or less  (2)

o Once a month or less  (3)

o A few times a month  (4)

o Once a week  (5)

o A few times a week  (6)

o Every day  (7)

Q11 I feel burned out from my work?

o Never  (1)

o A few times a year or less  (2)

o Once a month or less  (3)

o A few times a month  (4)

o Once a week  (5)

o A few times a week  (6)

o Every day  (7)

End of Block: Stress/Burnout

Start of Block: Competencies

Q12 Competencies Strongly agree (4)

Somewhat agree (5)

Neither agree nor disagree (6)

Somewhat disagree (7)

Strongly disagree (8)

Overall, I felt prepared for residency when I entered residency? (6)

o  

o  

o  

o  

o  

I was prepared to communicate effectively when I entered residency? (7)

o  

o  

o  

o  

o  

I was prepared professionally when I entered residency? (8)

o  

o  

o  

o  

o  

I was prepared to work effectively as a teammate when I entered residency? (9)

o  

o  

o  

o  

o  

I was prepared to make decisions with limited knowledge when I entered residency? (10)

o  

o  

o  

o  

o  

I was prepared to navigate difficult situations when I entered residency? (11)

o  

o  

o  

o  

o  

I possessed the efficiency needed to act effectively as a resident when I entered residency? (12)

o  

o  

o  

o  

o  

I was prepared to use consultation services when I entered residency? (13)

o  

o  

o  

o  

o  

I was prepared to take full responsibility of patients when I entered residency? (14)

o  

o  

o  

o  

o  

I was prepared to function effectively under stress when I entered residency? (15)

o  

o  

o  

o  

o  

Q23 Rank the the most important skill needed to succeed in Internship

o Communication  (1)

o Professionalism  (2)

o Efficiency  (3)

o Teamwork  (4)

o Decision-Making  (5)

o Knowledge/skills needed for Common Medical Problems  (7)

o Ability to consult successfully  (8)

o Other  (9) __________________________________________________

End of Block: Competencies

Start of Block: Elnicki, Varma, Chang, & WSU

Q24 __________ is a valuable component of a transition to residency course?

Strongly agree (2)

Somewhat agree (4)

Neither agree nor disagree (6)

Somewhat disagree (7)

Strongly disagree (8)

Sepsis and Shock (1)

o  

o  

o  

o  

o  

Venous Thromboembolism (2)

o  

o  

o  

o  

o  

Periprocedural Management (3)

o  

o  

o  

o  

o  

Acute renal failure, acid-base, and fluid management (4)

o  

o  

o  

o  

o  

Diabetes mellitus acute management (5)

o  

o  

o  

o  

o  

HIV complications (6)

o  

o  

o  

o  

o  

Acute cardiovascular disease (7)

o  

o  

o  

o  

o  

Palliative care (8)

o  

o  

o  

o  

o  

Transfusion (9)

o  

o  

o  

o  

o  

Common Infections (10)

o  

o  

o  

o  

o  

Q25 __________ is a valuable component of a transition to residency course?

Strongly agree (2)

Somewhat agree (3)

Neither agree nor disagree (4)

Somewhat disagree (5)

Strongly disagree (6)

Evidence-based medicine (1)

o  

o  

o  

o  

o  

Clinical Reasoning (2)

o  

o  

o  

o  

o  

Landmark articles review (3)

o  

o  

o  

o  

o  

Pharmacology review (4)

o  

o  

o  

o  

o  

Pain and Pain control (5)

o  

o  

o  

o  

o  

Immunology (6)

o  

o  

o  

o  

o  

Substance Abuse (7)

o  

o  

o  

o  

o  

Critical care physiology (8)

o  

o  

o  

o  

o  

Oncology (9)

o  

o  

o  

o  

o  

Toxicology (10)

o  

o  

o  

o  

o  

Pathophysiology connections to common conditions (11)

o  

o  

o  

o  

o  

Nutrition (12)

o  

o  

o  

o  

o  

Q26 __________ is a valuable component of a transition to residency course?

Strongly agree (1)

Somewhat agree (2)

Neither agree nor disagree (4)

Somewhat disagree (5)

Strongly disagree (6)

Procedures (lines, blood gas, suturing, lumbar puncture) (1)

o  

o  

o  

o  

o  

ACLS (2)

o  

o  

o  

o  

o  

Airway and ventilator management (3)

o  

o  

o  

o  

o  

Critical Care simulations (4)

o  

o  

o  

o  

o  

Physical exam skills (5)

o  

o  

o  

o  

o  

Electrocardiogram Reading (6)

o  

o  

o  

o  

o  

Radiology review (7)

o  

o  

o  

o  

o  

Q27 __________ is a valuable component of a transition to residency course?

Strongly agree (1)

Somewhat agree (2)

Neither agree nor disagree (4)

Somewhat disagree (5)

Strongly disagree (6)

Team building (1)

o  

o  

o  

o  

o  

Order and Note writing (2)

o  

o  

o  

o  

o  

Consulting (3)

o  

o  

o  

o  

o  

Safe transitions of care, sign-out, discharge (4)

o  

o  

o  

o  

o  

Teaching as an intern (5)

o  

o  

o  

o  

o  

Giving/Receiving Feedback (6)

o  

o  

o  

o  

o  

Duty Hours (7)

o  

o  

o  

o  

o  

Quality improvement and Patient Safety (8)

o  

o  

o  

o  

o  

Interactions with medical team (9)

o  

o  

o  

o  

o  

Common Nurse calls (10)

o  

o  

o  

o  

o  

Q28 __________ is a valuable component of a transition to residency course?

Strongly agree (1)

Somewhat agree (2)

Neither agree nor disagree (4)

Somewhat disagree (5)

Strongly disagree (6)

Presentation skills (1)

o  

o  

o  

o  

o  

Dealing with mistakes (2)

o  

o  

o  

o  

o  

Dealing with death (3)

o  

o  

o  

o  

o  

Discussing prognosis and code status (4)

o  

o  

o  

o  

o  

Difficult patients and families (5)

o  

o  

o  

o  

o  

Conflict resolution (6)

o  

o  

o  

o  

o  

Impaired collegues (7)

o  

o  

o  

o  

o  

Informed Consent (8)

o  

o  

o  

o  

o  

Language barriers and translator use (9)

o  

o  

o  

o  

o  

Q29 __________ is a valuable component of a transition to residency course?

Strongly agree (1)

Somewhat agree (2)

Neither agree nor disagree (4)

Somewhat disagree (5)

Strongly disagree (6)

What to expect from residency (1)

o  

o  

o  

o  

o  

Time Management (3)

o  

o  

o  

o  

o  

Work-life balance, coping with stress (4)

o  

o  

o  

o  

o  

Personal finances (5)

o  

o  

o  

o  

o  

Legal issues and mal practice (6)

o  

o  

o  

o  

o  

Career Development (7)

o  

o  

o  

o  

o  

High-value care (8)

o  

o  

o  

o  

o  

Health care economics, health care reform (9)

o  

o  

o  

o  

o  

Coding and billing (10)

o  

o  

o  

o  

o  

Q30 __________ is a valuable component of a transition to residency course?

Strongly agree (1)

Somewhat agree (2)

Neither agree nor disagree (3)

Somewhat disagree (4)

Strongly disagree (5)

LGBTQ patients (1)

o  

o  

o  

o  

o  

Smoking Cessation (2)

o  

o  

o  

o  

o  

Mentoring (3)

o  

o  

o  

o  

o  

How to respond from sexual harassments from patients (4)

o  

o  

o  

o  

o  

Telehealth (10)

o  

o  

o  

o  

o  

Oxygen Lines (11)

o  

o  

o  

o  

o  

End of Block: Elnicki, Varma, Chang, & WSU
